# Disseminated nocardiosis in an immunocompetent patient

**DOI:** 10.7196/AJTCCM.2019.v25i4.026

**Published:** 2019-12-06

**Authors:** M Ndlovu, P M Jeena, S A Thula, S A Singh, R Masekela

**Affiliations:** Department of Paediatrics and Child Health, Nelson R Mandela School of Clinical Medicine, University of KwaZulu-Natal, Durban, South Africa

**Keywords:** actinomycetales, disseminated infection, acid-fast bacteria, slow growing bacteria

## Abstract

Nocardiosis is a rare opportunistic bacterial infection. We describe an 8-year-old immunocompetent patient who presented with constitutional symptoms suggestive of probable tuberculosis (TB) in whom we confirmed a diagnosis of nocardiosis. *Nocardia* is a Gram-positive bacterium that is ubiquitous in soil and decaying vegetable matter. *N. asteroides* is the most common species. Despite the traditional description of nocardiosis as an opportunistic infection, case reports and case series of pulmonary nocardiosis have recently been reported in immunocompetent patients. Three clinical presentations of nocardiosis have been described; acute, subacute and chronic suppurative infections with episodes of exacerbations and remissions. We describe the presentation, diagnosis, management and prognosis of a rare case of disseminated nocardiosis managed initially as disseminated TB with no improvement.

## Case

**Fig. 1:**
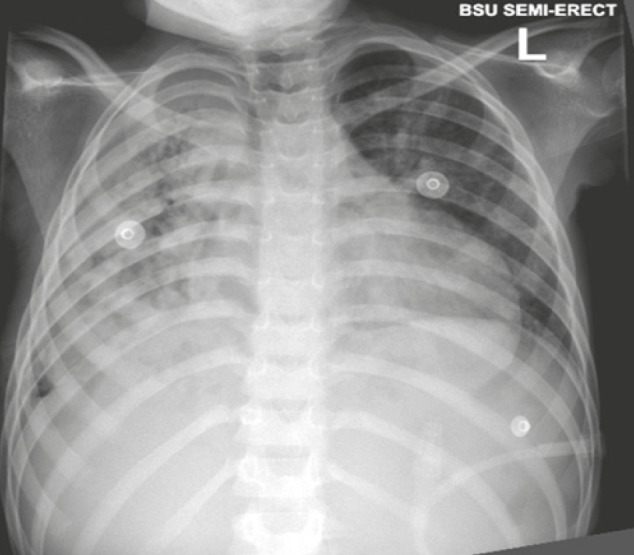
Right-sided consolidation with areas of breakdown indicative of a necrotising pneumonia

An 8-year-old female child was referred to Inkosi Albert Luthuli Central Hospital (IALCH) from a provincial hospital with a 4-month history of chronic purulent sputum, severe weight loss and constitutional symptoms. There was no tuberculosis (TB) contact but her 6-year-old brother had similar symptoms. They lived on a farm at Nkonjeni in rural KwaZulu-Natal, South Africa. She was assessed as having pulmonary tuberculosis and commenced on 4-drug anti-tuberculosis therapy (rifampicin/isoniazid/pyrazinamide and ethambutol) at the provincial hospital. The diagnosis of TB was based on the clinical presentation and chest radiology (Fig. 1).

GeneXpert and TB cultures were negative. The erythrocyte sedimentation rate (ESR) was greater than 140 mm/hr, monocytes 2.56 ×10^9^ /L and the globulin fraction was 37 g/L. Her HIV test was negative.

**Fig. 2:**
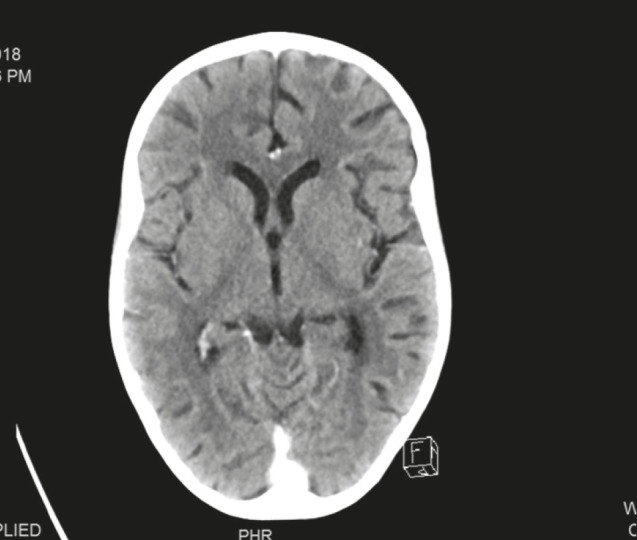
Computed tomography brain scan showing involution with brain atrophy with no abscesses

After 2 months of TB treatment, the patient deteriorated and presented to the provincial hospital with severe respiratory distress. A chest X-ray showed a right pleural effusion, a thoracentesis yielded pus and an inter-costal chest drain was inserted. She developed respiratory failure, and she was referred to the paediatric intensive care unit (PICU) at IALCH for mechanical ventilation. On examination, she was clinically wasted, weighed 24 kg (*z*-score between –2 and –3), was in respiratory distress, displaying pallor, clubbing and axillary lymphadenopathy. She had absent breath sounds on the right hemithorax with left-sided crackles. Abdominal examination revealed a 7 cm firm hepatomegaly with 3 cm splenomegaly. She was encephalopathic with meningism. An initial assessment of disseminated TB was made. She was intubated and ventilated. TB treatment was continued, and ethambutol was changed to ethionamide. Meropenem was added to the treatment. An urgent computed tomography (CT) brain scan showed age incongruent cerebral and cerebellar involutional changes with no basal enhancement or hydrocephalus or features of raised intracranial pressure (Fig. 2). Lumbar puncture showed lymphocytes of 28, neutrophils of 12, no erythrocytes, glucose 1.1 mmol/L, protein 2.5 g/L and chloride 122 mmol/L. Admission blood results: white blood cells were 45.3 ×10^9^ /L, with predominant neutrophilia of 35 ×10^9^ /L and monocytes of 4.99 ×10^9^ /L, Platelets were 1169 ×10^9^ /L, ESR >140 mm/hr and procalcitonin 52 ng/dL.

**Fig. 3:**
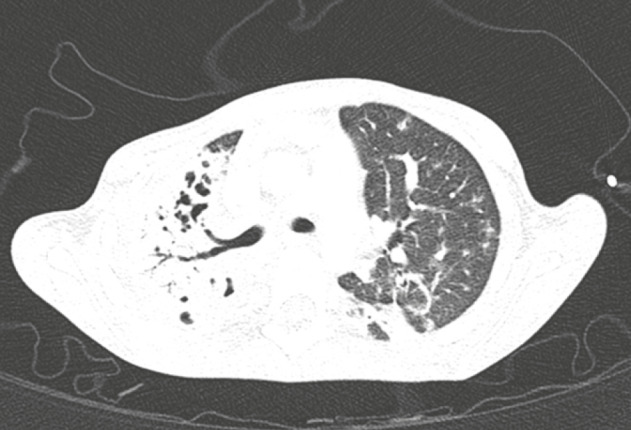
Computed tomography chest scan showing destruction of the entire right lung with bronchiectasis with cavitatory disease.

Gene Xpert and TB culture were negative. A CT chest scan showed destruction of the right lung with bronchiectasis and cavities (Fig. 3). Endotracheal aspirates and pleural fluid cultured *Nocardia* spp. sensitive to meropenem and co-trimoxazole. An assessment of disseminated nocardiosis was made. The patient was treated with meropenem and high-dose co-trimoxazole and after 7 days of therapy she showed signs of improvement, was successfully extubated and transferred to the infectious diseases unit to continue with both antibiotics and TB treatment. A month later, she developed generalised tonic-clinic seizures and decreased level of consciousness (Glasgow Coma Scale (GCS) score 7/15) and localising sign of dilated left pupil. She was then re-ventilated and transferred back to PICU. An urgent CT brain showed active tetra-ventricular hydrocephalus with transependymal seepage. An external ventricular drain was inserted by the neurosurgeons; however, the patient continued to deteriorate and demised on the ventilator.

Her 6-year-old brother was admitted to the infectious diseases unit with a persistent cough with no involvement of the central nervous system. His sputum cultured *Norcadia* spp. sensitive to imipenem, ceftriaxone and co-trimoxazole. He was treated with ceftriaxone and high-dose cotrimoxazole. He recovered completely and was discharged home.

## Discussion

The *Nocardia* genus of aerobic Gram-positive, branching, filamentous, weakly acid-fast bacteria cause a range of infectious diseases, including isolated pulmonary and skin infections, as well as disseminated disease.^[2]^ The organisms contain mycolic acids in their cell walls, and they are closely related to mycobacteria. *Nocardia* spp. are ubiquitous in soil and decaying vegetable matter. Eighty-eight species of *Nocardia* have been described and 46 cause disease in humans. The most common species are **N. asteroides** and *N. brasiliensis*. ^[3,4]^ Transmission in humans is by direct skin inoculation or inhalation.

Pulmonary *Nocardia*, the major site of presentation, is rare but potentially serious. It typically occurs in immunosuppressed patients but can be found in immunocompetent individuals.^[5]^ The lung manifestations include necrotising pneumonia, cavitatory disease and multiple lung abscesses or empyema.^[5]^ Co-infection with TB is rare; however, a few cases have been reported.^[5,6]^ The brain is the most common secondary site of dissemination. Brain abscesses and meningitis are the frequent central nervous system manifestations. The cerebrospinal fluid (CSF) picture resembles that of TB meningitis with high protein and lymphocytic predominance. ^[7]^ The radiological features of nocardiosis can be non-specific. In a small study by Lyu *et al*.,^[8]^ chest X-ray changes encompassed consolidation, ground-glass opacities (GGOs), empyema and cavities. Multifocal ring-enhancing lesions were the dominant features of brain and muscle nocardiosis.^[8]^

Diagnosis is confirmed by isolating *Nocardia* from respiratory secretions, skin biopsies, aspiration from deep collections, CSF and biopsy smears. Gram staining and modified Kinyoun staining are the techniques for the identification of *Nocardia*. The colonies have a chalky white or cotton ball appearance because of the presence of abundant aerial filaments.^[7]^ *Nocardia* species are slow growing so the laboratory should set up cultures for a prolonged duration of up to 3 weeks.^[4]^ *Nocardia* displays variable *in vitro* antimicrobial susceptibility patterns, and management of *Nocardia*l infections must be individualised. Sulphonamides such as co-trimoxazole have been the mainstay of treatment for nocardiosis but newer studies indicate resistance to co-trimoxazole.^[3]^

Combination therapy of co-trimoxazole plus a carbapenem, or third-generation cephalosporins, or minocycline with or without amikacin have been the primary regimens for the treatment of pulmonary, disseminated, or brain nocardiosis especially in patients receiving immunosuppressive agents.^[9]^

Despite appropriate therapy, the overall mortality rate is high due to delay in diagnosis or to the debilitated state of patients with severely compromised host defences. Wang *et al*. ^[3]^ showed that the mortality from lung nocardiosis in their cohort was 33%.

## Conclusion

This case describes a rare but serious chronic bacterial infection that presents similarly to tuberculosis. *Nocardia* infection must be suspected in both immunocompromised and immunocompetent patients with suspected TB who are unresponsive to anti-tuberculous treatment. Timeous diagnosis and initiation of appropriate therapy is crucial in order to decrease the high mortality associated with *Nocardia* sepsis.
